# Supplementary material for: Trypsin and Trypsinogen Activation Peptide in the Prediction of Severity of Acute Pancreatitis
Source: Life (Basel). 2024 Aug 23;14(9):1055. doi: 10.3390/life14091055 (PMC11433092; doi:10.3390/life14091055)
Supplement: Supplementary file 1 [file life-14-01055-s001.zip › life-2929890-supplementary.pdf]

Trypsin and Trypsinogen Activation Peptide in the Prediction of Severity of Acute Pancreatitis

Andreas Allemann [1,†], Sebastian M. Staubli [2,*,†] and Christian A. Nebiker [3]

1 Department of Psychiatry Biel, PZM AG, Vogelsang 84, 2501 Biel, Switzerland; andreas.allemann@pzmag.ch
2 HPB and Liver Transplantation Service, Royal Free London NHS Foundation Trust, Pond Street, London NW3 QG, UK
3 Department of Surgery, Cantonal Hospital Aarau, Tellstrasse 25, 5001 Aarau, Switzerland; christian.nebiker@ksa.ch
* Correspondence: s.staubli@nhs.net
† These authors contributed equally to this work.

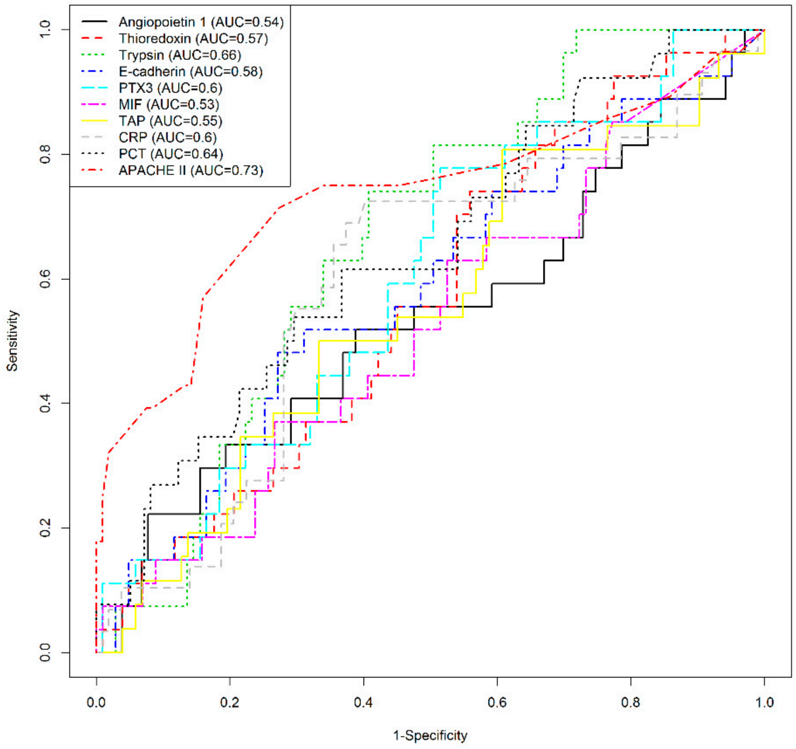

Figure S1. Discriminatory ability of the biomarkers to predict organ failure (defined according to the modified Marshall score) or death. (A) Among all patients. (B) Among patients without organ failure at study inclusion.
